# Spontaneous lumbar artery rupture and massive retroperitoneal hematoma, successfully treated with arteriographic embolization

**DOI:** 10.12669/pjms.35.2.639

**Published:** 2019

**Authors:** Jin Yong Kim, Song Am Lee, Jae Joon Hwang, Jae Bum Park, Sang Woo Park, Yo Han Kim, Hyeong Ju Moon, Woo Surng Lee

**Affiliations:** 1 *Jin Yong Kim, M.D. Department of Emergency Medicine, School of Medicine, Konkuk University, Konkuk University Chungju Hospital, Chungju-si, Chungbuk, Republic of Korea*; 2 *Song Am Lee, M.D., Ph.D. Department of Thoracic and Cardiovascular Surgery, School of Medicine, Konkuk University, Konkuk University Seoul Hospital, Seoul, Republic of Korea*; 3 *Jae Joon Hwang, M.D., Ph.D. Department of Thoracic and Cardiovascular Surgery, School of Medicine, Konkuk University, Konkuk University Seoul Hospital, Seoul, Republic of Korea*; 4 *Jae Bum Park, M.D., Ph.D. Department of Thoracic and Cardiovascular Surgery, School of Medicine, Konkuk University, Konkuk University Seoul Hospital, Seoul, Republic of Korea*; 5 *Sang Woo Park, M.D., Ph.D. Department of Radiology, School of Medicine, Konkuk University, Konkuk University Seoul Hospital, Seoul, Republic of Korea*; 6 *Yo Han Kim, M.D., Ph.D. Department of Thoracic and Cardiovascular Surgery, School of Medicine, Konkuk University, Konkuk University Chungju Hospital, Chungju-si, Chungbuk, Republic of Korea*; 7 *Hyeong Ju Moon, R.N. Department of Thoracic and Cardiovascular Surgery, School of Medicine, Konkuk University, Konkuk University Chungju Hospital, Chungju-si, Chungbuk, Republic of Korea*; 8 *Woo Surng Lee, M.D., Ph.D. Department of Thoracic and Cardiovascular Surgery, School of Medicine, Konkuk University, Konkuk University Chungju Hospital, Chungju-si, Chungbuk, Republic of Korea*

**Keywords:** Angiography, Embolization, Hypovolemic Shock, Lumbar Artery, Retroperitoneal Hematoma

## Abstract

**Background and Objective:**

Massive retroperitoneal hematoma caused by lumbar artery rupture is generally associated with trauma or retroperitoneal malignancy. However, despite recent advances in technologies and tools, spontaneous lumbar artery rupture is a very rare disease entity but remains a challenging problem because it is frequently associated with significantly high mortality and morbidity and is very difficult to make a correct diagnosis.

**Methods:**

We evaluated the databases of the PubMed, Embase, Cochrane Central Register of Controlled Trial, Google Scholar, the KoreaMed and the Research Information Sharing Service databases, and a detailed systematic review was performed by searching in PubMed. The initial search was performed on 3 February 2018 and a second search conducted in 29 January 2019.

**Results:**

A total of 10 case reports on massive hemoperitoneum caused by spontaneous lumbar artery rupture were identified. Of the 10 case reports involving 14 patients, eight were male and six were female under 62.71 ± 13.93. Of the 14 patients, 9 (64.3%) surviving with transcatheter arterial embolization, three (21.4%) died of multi-organ failure or hypovolemia, and two (14.3%) had no definite records on survival or death.

**Conclusions:**

A massive retroperitoneal hematoma caused by lumbar artery rupture should be considered in patients with late-onset shock accompanied by blunt abdominal/pelvic trauma. Furthermore, early detection and urgent embolization would prevent further complications and eliminate the need for surgical interventions.

## INTRODUCTION

Retroperitoneal bleeding occurs following lumbar artery injury due to severe trauma or surgical intervention.^1^ Massive retroperitoneal hematoma has been shown to develop after rupture of underlying abdominal aortic aneurysm or malignancies of the retroperitoneal organs such as the kidney and the adrenal gland.^2^ Spontaneous lumbar artery rupture (LAR) is an extremely rare disease entity, and there is still a lack of information on its exact incidence and underlying mechanism. This disease entity has been increasing due to frequent antiplatelet or anticoagulant therapy^3^, and previous studies have reported that this entity is related to undetected vascular lesion, coagulopathies^4^,^5^ and hemodialysis.^6^ However, LAR very rarely occurs in patients who have neither underlying disease nor history of anticoagulant therapy. This report describes a case of spontaneous LAR and discusses its clinical presentation, diagnosis and treatment.

## METHODS

We systematically scrutinized the PubMed, Embase, Cochrane Central Register of Controlled Trial, Google Scholar, the KoreaMed and the Research Information Sharing Service databases, and a detailed systematic review was performed by searching in PubMed using the following keywords: “lumbar artery” AND “rupture” AND “retroperitoneal hematoma” AND “spontaneous” AND/OR “angiography” AND/OR “surgery” AND/OR “shock” AND/OR “bleeding” AND/OR “anticoagulation”. Only 10 case studies with a short review were identified, and we could not find any comparative studies or randomized clinical trials. Studies were also selected based on the following exclusion criteria, and these criteria were as follows: (1) complication by surgical approach, such as spine discectomy, fusion, transplantation, or endovascular aortic repair; (2) underlying aorta lesion, such as abdominal aortic aneurysm, pseudoaneurysm, or renal artery aneurysm; (3) direct traumatic injury; (4) venous complication by pelvic varix rupture or aorta-vein fistula; (5) complication by procedure, such as cardiac catheterization or renal biopsy; (6) unusual underlying medical condition, such as carcinoma, malignancy or metastatic lesion. The initial search was performed on 3 February 2018 and a second search conducted in 29 January 2019.

### Presentation case

A 92-year-old man presented to our emergency department after a motorcycle crash with a 25-ton dump truck. He had no remarkable history of specific disease or operation except for >24 years of well controlled hypertension with calcium channel blockers at a regional hospital. In his youth, he was a professional soldier for 20 years, a farmer for the rest of his life, and was without any definite job for the past several years. At presentation, his mental state was normal, he had no loss of consciousness, and the Glasgow coma scale score was 15 points. His vital signs were as follows: blood pressure (BP), 79/44 mm Hg; pulse rate (PR), 96 beats/min; respiratory rate (RR), 25 times/min; body temperature (BT), 36.7°C; and oxygen saturation measured using a pulse oximeter, 91%. Physical examination revealed tenderness on the right lower thorax, without definite tenderness or rebound tenderness on the abdomen and the pelvis. Extended focused assessment with sonography for trauma was performed in the thorax and the abdomen, and revealed neither definite intra-abdominal fluid collections nor thorax lesions, except for a right-sided small pneumothorax. No objective sensory or motor disturbance in either of the upper or lower extremities was observed, and distal upper and lower limb pulses were normal, except for right humerus fracture. After initial resuscitation and management, vital signs were as follows: BP, 86/55 mm Hg; PR, 65beats/min; RR, 20times/min; BT, 36.7°C; SpO_2_, 93%. Laboratory tests revealed WBC, 12,500/μ1 and hemoglobin 13.4 g/dl. Initial chest plain radiographs as well as brain, chest, abdomen and pelvis computed tomography (CT) scans with intravenous contrast media revealed the following findings: right-sided multiple rib fractures (7, 8, 9, 10, 11 and 12th); right-sided traumatic hemopneumothorax and lung contusion; right humerus shaft and scapular fracture; right ear laceration; cerebral concussion without intracranial hemorrhage; and compression fracture of the 12th thoracic spine body and hypovolemic shock. Closed thoracostomy drainage with a 24-Fr thoracic catheter was performed to resolve right traumatic hemopneumothorax, and more than 600mL of bloody color fluid and active persistent air leak gushed out through the thoracic tube.

**Fig.1:**
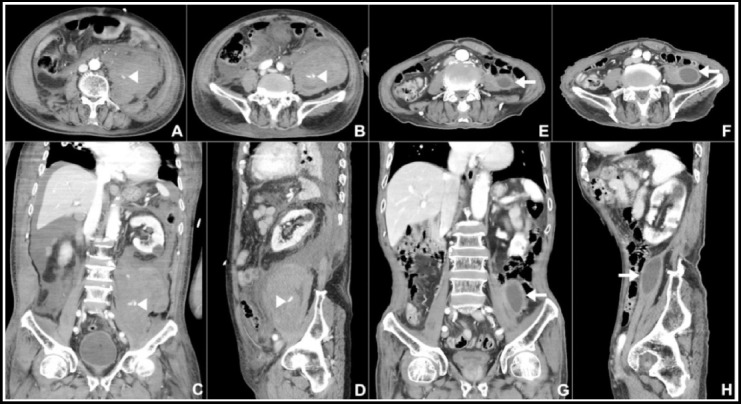
Transverse (A&B), coronal (C) and sagittal (D) enhanced abdomen and pelvis scans show a massive hematoma and extravasation of contrast dye into the retroperitoneal space. White arrow head indicates active lumbar arterial bleeding and a preformed massive hematoma in the retroperitoneal space. Seventy-two days after transcatheter arterial embolization, transverse (E&F), coronal (G) and sagittal (H) enhanced abdomen and pelvis scans show a resolving hematoma. White arrow indicates a 3.0-cmresolving hematoma in the retroperitoneal space.

**Fig.2:**
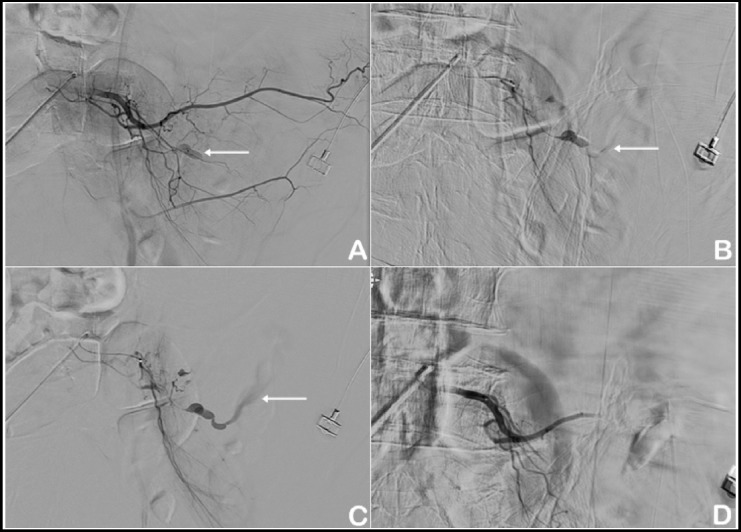
A. Angiography was performed through the right common femoral artery, a 5-Fr catheter was inserted, and the left 4th lumbar artery was selected. Initial angiogram shows dye leakage and pseudoaneurysmal change in a branch of the left lumbar artery (white arrow). B & C. Delayed angiogram of a ruptured lumbar artery branch taken after super selection with a microcatheter (1.9-Fr) shows more prominent extravasation of dye into the retroperitoneal space. White arrow indicates active dye leakage and pseudoaneurysmal change. D. Angiogram taken after embolization shows successful embolization without any dye leakage.

He was first admitted to the intensive care unit and closely treated for the control of hypovolemic shock, anemia, thrombocytopenia and disseminated intravascular coagulation. On the sixth day in the intensive care unit, follow-up laboratory tests and CT scans confirmed improved state from the initial lesions, and he was transferred to the general ward. Then, he resumed a normal diet and showed improvement in daily life activities. On the 12^th^ hospital day, he underwent open reduction and intramedullary nailing for right humerus shaft fracture by an orthopedic surgeon under general anesthesia, and the operation was smoothly progressed without any events. Ninety minutes after general anesthesia, an anesthesiologist suddenly encountered slight difficulty in ventilator bagging, BP dropping and distension of the abdomen. The BP dropped to 60/35 mmHg and PR increased to 125 beats/min. Laboratory tests revealed a hemoglobin level of 6.5 g/dl suggestive of hypovolemic shock, and he received fluid challenge and transfusion. After completion of the surgery, he was re-evaluated with contrast-enhanced abdomen-pelvis CT, which revealed a massive retroperitoneal hematoma and active contrast-dye extravasation within the hematoma not seen on initial and follow-up abdomen-pelvis CT scans (Fig.1). Urgent angiography was performed under local anesthesia through the right common femoral artery, and 5-Fr Cobra, Omni and Shepherd hook catheters were sequentially inserted. The dye leakage was observed at the initial phase, and extravasation into the retroperitoneal space was noted at the delayed phase. The meticulous angiographic approach showed a dye leak and a pseudoaneurysmal change in a branch of the left fourth lumbar artery. The left fourth lumbar artery was selected, and delayed angiograms after superselection with a microcatheter (1.9-Fr) showed more prominent extravasation of dye into the retroperitoneal space on a ruptured left fourth lumbar artery branch. The ruptured artery was successfully embolized using a 1:1 mixture of histoacryl (monomeric n-butyl-2-cyanoacrylate) and lipiodol (ethiodized oil) (Fig.2). After successful embolization, his vital signs were dramatically stabilized. Due to newly onset disseminated intravascular coagulation and acute respiratory distress syndrome, he was managed in the intensive care unit for 60 days. The patients under went tracheostomy on the 28^th^ hospital day, with the ventilator successfully weaned on the 70^th^ hospital day, and was transferred again to the general ward on the 72^th^ hospital day. He was discharged from the hospital without any sequelae and further events after full recovery from the massive bleeding from lumbar artery, hypovolemic shock, disseminated intravascular coagulation and acute respiratory distress syndrome.

## RESULTS

The detailed informations in these reports are summarized in Table-I. We identified a total of 10 case reports on spontaneous LAR in 14 patients. Of the 10 case reports involving 14 patients, eight were male and 6 were female under 62.71 ± 13.93 (mean age ± standard deviation). On the time of spontaneous LAR and presentation, nine patients had received hemodialysis therapy and the remaining 5 received anticoagulation, antiplatelet and heparin. Of the 14 patients, nine (64.3%) surviving with transcatheter arterial embolization, three (21.4%) died of multi-organ failure or hypovolemia, and two (14.3%) had no definite records on survival or death. Massive retroperitoneal hematoma due to spontaneous LAR was reported in two patients who needed anticoagulation with Nafamostat mesilate for continuous renal replacement therapy such as hemodialysis or hemofiltration.

**Table-I:** Systematic review of the literature massive retroperitoneal hematoma, which were caused by spontaneous lumbar artery rupture and treated with arteriographic embolization.

Year	Reporter	No	Sex/Age	Risk factors	RRT	Tx	Mortality	COD
2018	Present case	1	M/92	HTN, advanced age	None	TAE	SV	-
2017	Hwang NK et al.^6^	3	M/43	DM, LC, HTN, CRF, HDHP	HD	TAE	SV	-
			F/69	DM, CAD, CRF, HDHP, ASP, CLO	HD	TAE	NS	HS, MOF
			M/48	WF, PAOD, CS, CRF, HDHP, CAD, CLO, PLE, enoxaparin	PD, HD	TAE	SV	-
2015	Park JK et al.^14^	1	M/71	HP, DM, pulmonary edema	CRRT	TAE	SV	-
2014	Yamamura et al. 11	3	M/45	NM, LC, s/p LT	CRRT	TAE	SV	-
			M/63	NM, fulminant hepatitis	CRRT	TAE	NS	MOF
			F/77	HP, post cardiac arrest syndrome, thyroid carcinoma	None	TAE	SV	-
2011	Surani S et al.^15^	1	M/67	ESRD on HD, DM, CAD, MVR , WF	HD	TAE	SV	-
2009	Sun PL et al.^16^	1	F/73	Enoxaparine, s/p THR	None	TAE	SV	-
2007	Fortina M et al. 4	1	M/78	Fondaparinux, s/p THR, s/p NPT	None	TAE	SV	-
2004	Hama Y et al.^17^	1	F/38	Alcoholic LC	None	TAE	NI	-
2004	Pyo SH et al.^9^	1	F/57	CRF, HDHP, HTN, CLO	HD	TAE	NI	-
2003	Schuster F et al.^18^	1	M/77	BC, anticoagulation, s/p HVR	None	TAE	SV	-
2001	Halak M et al.^8^	1	F/72	CRF, HDHP, HTN, CHF, IHD	HD	TAE	NS	HS, MOF

Total	11 reports	15	M/F:9/6	62.71 ± 13.93			SV/NS/NI,10/3/2	

***Abbreviations:*** No: number, RRT: Renal replacement therapy, Tx: Treatment, M: Male, F; Female, COD: Cause of death, HD: Hemodialysis, DM: Diabetes mellitus, LC: Liver cirrhosis, HTN: Hypertension, CRF: Chronic renal failure, HP: Heparin, HDHP: Hemodialysis with heparin, TAE: Transcatheter arterial embolization, SV: Survival, NS: Non-survival, ASP: Aspirin, CLO: Clopidogrel, HS: Hypovolemic shock, MOF: Multiple organ failure, PD: Peritoneal dialysis, WF: Warfarinization, PAOD: Peripheral artery occlusive disease, CS: Cerebral stroke, CAD: Coronary artery disease, PLE: Pletaal (cilostazol), NM: Nafamostat mesilate, LT: Liver transplantation, ESRD: End-stage renal disease, MVR: Mitral valve replacement, S/P: Status post, THR: Total hip replacement, NPT: Nephrectomy, NI: Not identified, BC: Bladder carcinoma, HVR: Heart valve replacement, CHF: Congestive heart failure, IHD: Ischemic heart disease,

## DISCUSSION

Retroperitoneal hematoma frequently results from retroperitoneal neoplasms in the kidney and the adrenal gland as well as abdominal aortic aneurysm, traumatic vascular injury and coagulopathy. It can occur in the absence of underlying causes including trauma or malignancy, and several cases have been reported in renal failure patients on hemodialysis involving anticoagulant agents.^1^,^2^,^6^ However, spontaneous LAR is a quite rare disease entity, even in chronic kidney disease patients. In 1998, Kalinowski and Trerotola^7^ reported a case of retroperitoneal hematoma developed after cardiac catheterization, as a complication of the puncture, so that this case was excluded from the literature review. In 2001, Halak M et al.^8^ presented the first case of spontaneous LAR in a 72-year-old female chronic kidney disease patient on hemodialysis. In this case, hemodialysis on heparin was a main risk factor for spontaneous LAR, and urgent transcatheter arterial embolization saved from further bleeding. Unfortunately, the patient died of multiorgan failure and hypovolemia. Pyo SH et al.^9^ reported spontaneous and simultaneous rupture of the lumbar and inferior epigastric arteries in a 57-year-old female chronic kidney disease Korean patient during hemodialysis. Hemodialysis on heparin and clopidogrel were also a main risk factor for spontaneous LAR, and urgent transcatheter arterial embolization stopped further blood loss.

Major risk factors for spontaneous LAR include advanced age, renal insufficiency and anticoagulant therapy; half of the patients show clinical characteristics of renal dysfunction.^3^,^6^ The etiologies and mechanisms involved in spontaneous LAR are known to be multifactorial, among which reduced adhesiveness and aggregation of platelets, lowered activity of platelet factor III, and impaired prothrombin consumption are the most common causes.^10^ Yamamura M et al.^11^ have suggested that muscle strain can induce LAR. Patients with retroperitoneal hematoma show a mortality of 50%, which is mainly attributed to anticoagulant or antiplatelet therapy. The typical clinical features of retroperitoneal hematoma include abdominal pain, hypotension, and subsequent development of abdominal distention and mass.^6^ Enhanced abdomen and pelvis CT is essential for the evaluation of spontaneous LAR, which identifies homogeneous and relatively high-density contrast-enhanced masses in the retroperitoneal space. In patients with a retroperitoneal mass due to spontaneous LAR, urgent angiography is essential to perform transcatheter arterial embolization after confirmation of active bleeding focus. In recent publication, Kim and Lee^12^ reported similar hypovolemic shock patient caused by delayed-onset superior gluteal artery rupture, and successfully treated with arteriographic embolization. Patients showing active contrast leakage on angiography are difficult to manage with conservative treatment and thus are indicated for transcatheter arterial embolization.^3^,^6^ Initial conservative treatment of LAR patients with hypotension includes crystalloid resuscitation and transfusion with packed red cells, fresh frozen plasma and platelet concentrate for the correction of coagulopathy. Surgical evacuation is restrictively recommended in patients who do not improve after transcatheter arterial embolization, and should be considered in patients with serious cardiac output impairment, respiratory insufficiency, nephropathy associated with oliguria and acute renal injury, untreated hypotension, or ischemia of the intestine.^3^

Anatomically, the first to fourth lumber arteries arise from the dorsal aspect of the abdominal descending aorta usually at the level of the transverse process; however, the fifth lumber artery arises from the middle sacral artery. The lumber arteries run lateral ward on the bodies of the lumbar vertebrae and form a network of lumber arterial collaterals. They anastomose with the intercostal, subcostal, superior/inferior epigastric and iliolumbar arteries. They supply the lumbar vertebrae, muscles of the back and posterior abdominal wall.^13^ In a published report, excessive muscular strain could be a hypothetical mechanism for LAR; however, the exact mechanism for spontaneous LAR is not elucidated.^11^

## CONCLUSION

Spontaneous LAR bleeding should be considered in hypovolemic shock associated with hemodialysis or anticoagulation. Additionally, early detection and embolization not only avert further complications such as multiorgan failure and hypovolemic shock, but also minimize the need for surgical interventions. Especially in a chronic renal failure patient with hemodynamic instability and no definite trauma or injury, spontaneous occult bleeding should be considered because the retroperitoneal space is a possible site of occult bleeding. The morbidity and mortality of spontaneous LAR can be lowered by early detection and proper treatment such as transcatheter arterial embolization.

### List of abbreviations

**LAR:** Lumbar artery rupture, **BP:** Blood pressure, **PR:** Pulse rate,

**RR:** Respiratory rate, **BT:** Body temperature, **CT:** computed tomography.

### Author`s Contribution

**JYK, WSL, YHK, SAL and HJM** conceived of and designed the study, collected and interpreted the data, and drafted the manuscript.

**WSL and JBP** collected and interpreted the data.

**SAL and WSL** analyzed and interpreted the data, and performed statistical analyses.

**WSL and JJH** participated in the study design, and revised the manuscript.

**WSL** critically reviewed the manuscript.

**SWP** performed Transcathetere arterial embolization.

All authors have read and approved the final manuscript and agree to be accountable for all aspects of the work.
